# Usability and Robustness of the Wixela Inhub Dry Powder Inhaler

**DOI:** 10.1089/jamp.2020.1603

**Published:** 2021-04-08

**Authors:** Richard Allan, Kelly Canham, Roisin Wallace, Dave Singh, Jon Ward, Andrew Cooper, Claire Newcomb

**Affiliations:** ^1^ Mylan, Inc., Sandwich, Kent, United Kingdom.; ^2^ Mylan Global Device Development, Dublin, Ireland.; ^3^ Medicines Evaluation Unit, The University of Manchester, Manchester University NHS Foundation Trust, Manchester, United Kingdom.

**Keywords:** asthma, COPD, device orientation, fluticasone propionate/salmeterol

## Abstract

***Background:*** Wixela Inhub is a generic version of Advair Diskus recently approved by the U.S. Food and Drug Administration. The Inhub inhaler delivers fluticasone propionate (FP)/salmeterol in a dry powder formulation. The goals of our studies were to demonstrate that the Inhub inhaler can be used by representative end users and confirm the robustness of the Inhub inhaler.

***Methods:*** Study 1: A nondosing usability assessment, the device orientation study, confirmed that intended users (represented by patients diagnosed with asthma or chronic obstructive pulmonary disease [COPD] who were naive to dry powder inhalers and current Advair Diskus users) could use the Inhub inhaler safely and effectively. Subjects were provided with an Inhub inhaler in commercial packaging, including instructions for use, and were asked to undertake three dose simulations using the inhaler. Subjects were encouraged to interact with this new drug delivery device as they would at home. Subjects were not provided with training on the use of the device. Subjects were observed interacting with the Inhub inhaler, and those who currently use Diskus were also observed interacting with the Diskus to determine whether their mental model of the use of Diskus impacted their interaction with the Inhub device, this assessment was not a primary outcome of the study. Study 2: This is an open-label clinical study to confirm the robustness of the Inhub inhaler after at home patient use. Subjects diagnosed with asthma or COPD were provided Inhub inhaler training and subsequently self-administered 3 weeks of twice daily doses of Wixela Inhub 250 μg FP/50 μg salmeterol in the home environment. The Inhub inhalers were returned to the investigator after ∼3 weeks of outpatient use for *in vitro* tests on the drug remaining in each inhaler.

***Results:*** Study 1 enrolled 110 subjects, and all completed the study. Most subjects (100/110) held the Inhub inhaler in the correct orientation and of those who did not, 9 still achieved a peak inhalation flow rate of ≥30 L/min and a total inhaled volume of ≥1 L, thus meeting the requirements of the study success criteria. In Study 2, 111 pediatric, adult, and elderly subjects with asthma or COPD received the study drug. After ∼3 weeks of outpatient use of the Inhub inhaler by subjects, comprehensive *in vitro* testing demonstrated that the FP and salmeterol pharmaceutical performance in the Inhub inhaler was preserved.

***Conclusions:*** The majority of subjects demonstrated safe and effective use of the Inhub inhaler. *In vitro* testing and inspections confirmed the robustness of the Inhub inhaler after outpatient use.

Clinical trial registration number: NCT02474017

## Introduction

For decades, inhaled therapy has been used successfully as the primary means to treat subjects with lung diseases such as asthma and chronic obstructive pulmonary disease (COPD). Aerosol formulations of medications provided through inhaler devices allow for noninvasive delivery, maximal pulmonary specificity, and rapid onset of effect, with minimal undesirable systemic effects.^(1–3)^ Current guidelines recommend inhaled corticosteroids (ICSs) and long-acting bronchodilators to control asthma and COPD, respectively, to reduce symptoms and decrease the likelihood of an exacerbation.^(4,5)^ For asthma, an ICS is generally recommended as an initial controller medication and, when an ICS alone is deemed insufficient, an inhaler combining an ICS and long-acting bronchodilator is often recommended.^(4)^ For COPD, long-acting bronchodilators are generally the recommended treatments, with an ICS added when symptoms/exacerbations persist despite optimal bronchodilation.^(5)^

The original portable aerosol-generating device developed in the 1950s was the pressurized metered-dose inhaler (pMDI).^(2,6)^ However, early metered-dose inhaler (MDI) devices required significant coordination by the patient to successfully deliver the appropriate dose into the lungs, and also utilized ozone-depleting chlorofluorocarbon as its propellant. Together, these factors prompted not only improvements to MDI devices but also the development of alternatives, including the dry powder inhaler (DPI).^(6,7)^ With a DPI, the patient's inspiration aerosolizes the powder contained in the inhaler, thus enabling oral inhalation of the drug, making it unnecessary for the patient to coordinate inhalation and drug release. Other recent improvements present in second-generation DPIs include multidose or multiunit devices in which individual premetered doses are provided by the manufacturer.^(2,3,6)^ Today, DPI devices represent compact, portable, and breath-actuated inhaler instruments that demonstrate higher lung deposition than pMDIs and contain no propellants.^(8)^

Advair Diskus (GlaxoSmithKline) is a widely prescribed ICS/long-acting β-agonist (LABA) combination drug (fluticasone propionate [FP]/salmeterol [FPS]) for subjects with asthma not controlled with ICS alone, and for subjects with COPD at high risk of exacerbations.^(4,5)^ With the expiration of the U.S. patent for Advair Diskus in 2016, several generic versions are currently advancing toward regulatory approval.^(9–12)^ The most advanced of these in development/approval is Wixela Inhub, composed of FPS inhalation powder (MGR001; Mylan) predispensed in a multidose inhaler (Inhub, CRC749; Mylan). An abbreviated new drug application for Wixela Inhub was recently approved by the U.S. Food and Drug Administration (FDA).

**FIG. 1.**
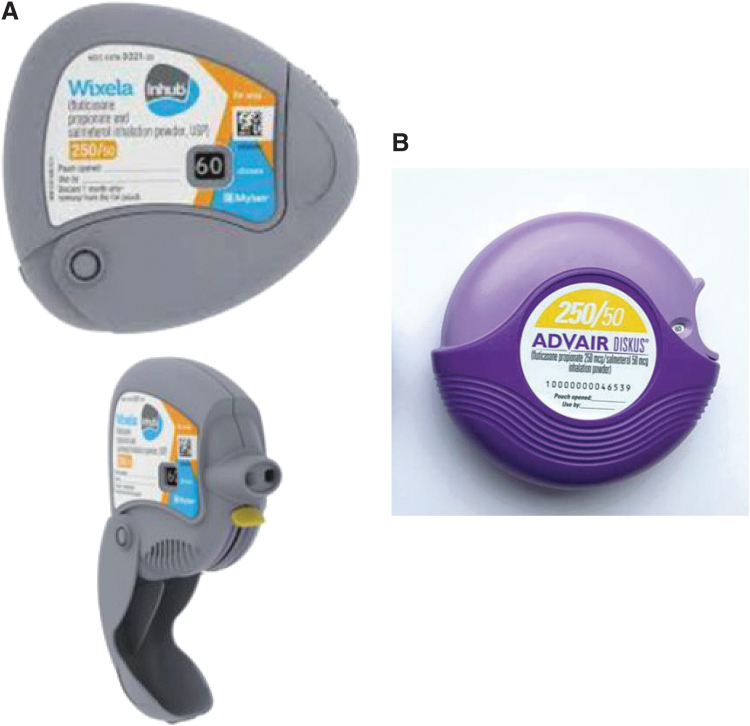
**(A)** Inhub Inhaler. **(B)** Diskus Inhaler.

The Inhub inhaler (Fig. 1A) is a small round handheld inhalation device that delivers FPS combination in a dry powder formulation. Inhub holds 60 doses and includes a dose counter to indicate how many doses remain. The Inhub inhaler is operated by completing four key steps: open the mouthpiece, push down a lever, inhale, and close the mouthpiece (“Inhub inhaler instructions for use”). These usage steps are also similar to those for the Diskus inhaler (Fig. 1B), with two primary differences: (1) to release a dose into the holding chamber, a lever is pushed down on the Inhub inhaler, whereas users slide a lever around the perimeter of the Diskus inhaler and (2) the Inhub inhaler is held vertically during inhalation, whereas the Diskus inhaler is held horizontally.

The FDA regulatory requirements for approval of drug delivery devices differ from those for stand-alone drug product medications.^(13,14)^ Specifically, it must be demonstrated that the device can be used safely and effectively by its intended users. As per the draft guidance on FP/salmeterol xinafoate 2013, generic Advair Diskus must demonstrate, as part of a weight of evidence approach, *in vitro* pharmaceutical equivalence and systemic pharmacokinetic bioequivalence along with local (lung) therapeutic equivalence that, in total, demonstrate therapeutic equivalence to Advair Diskus. In addition, generic Advair Diskus guidance requires demonstration of in-patient robustness, as well as *in vitro* verification of device robustness, to confirm that patients can use the device at home.

In this study we present the results of two studies: (1) a usability validation study to demonstrate that representative end users (Advair Diskus users, and those who never used a DPI) can use the Inhub inhaler safely and effectively to deliver a therapeutic dose, without training and with specific focus on orientation of use, and (2) an open clinical study to confirm the robustness of the Inhub inhaler (demonstrated by *in vitro* pharmaceutical performance of FP and salmeterol) after 3 weeks of twice-daily (BID) dosing of Wixela Inhub 250/50.

## Materials and Methods

### Study 1: usability validation study of Inhub inhaler

#### Study design and conduct

The study protocol for Study 1 was developed with feedback from the FDA and used simulated-use scenarios to demonstrate how subjects handle the Inhub device, with a focus on the orientation of the hold, and to ensure safe and effective use. The study protocol, subject recruitment, and test materials were reviewed and approved by an institutional review board. Subjects ≥18 years of age provided written informed consent, and those <18 years provided written informed assent and their guardians provided written informed consent that included consent to the recording of the session. Inhalers used in this study did not contain drug product; Inhub inhalers were assembled with empty dose pockets whereas Diskus inhalers were disassembled, the dose strip removed and replaced with an empty dose strip and reassembled. These actions do not impact the key operating features of either inhaler.

#### Study subjects

Study 1 enrolled two groups of subjects: those who were current Advair Diskus users and those who had never used a DPI (termed “DPI naive”). Subjects were further segmented into adolescent (≥12 to <18 years), adult (≥18 to <65 years), and elderly (≥65 years) age groups. Enrolled subjects were required to be diagnosed with asthma, COPD, or both, and Advair Diskus users must have been using the inhaler regularly for ≥3 months. The subject sample was required to have an even gender split to represent the gender percentage in the U.S. population (∼50% male/50% female ±10%), 5%–10% of subjects with limited manual dexterity or grip-strength issues, and ∼10% of subjects who were left handed. Manual dexterity/grip strength issues were identified by subjects' self-reported response to the following screening question: “Do you (or your child) have any manual dexterity issues in your hands or finger such as arthritis, joint pain, fibromyalgia, Parkinson's disease, or diabetic neuropathy or have significant difficulty grasping, opening, or manipulating objects?” The literacy level of each subject was recorded using the Slosson Oral Reading Test.

#### Study assessments

The study was conducted in research centers that could mimic a home-use environment. A moderator (or moderator and parent, for adolescent users) was present in the session room during assessments. Subjects were provided with an empty Diskus or Inhub inhaler in packaging representative of the commercial packaging, including the instructions for use (IFU), without training or instruction, and were encouraged to use the inhaler as they would if they had been prescribed a new drug delivery device and were using it at home. Subjects were monitored interacting with the inhaler by observers in an adjacent room separated by a one-way mirror, and all testing sessions were recorded for remote viewing and later analysis.

Each usability assessment session comprised three tasks (three simulated dose inhalations) with the Inhub inhaler. Advair Diskus users were asked to undertake a further three tasks (simulated dose inhalations) using an empty Diskus inhaler, the purpose of which was to assess compliance with the intended use of their current inhaler. At the start of the session, all subjects were given the inhaler and asked to take a dose. Subjects were given as much time as they needed to interact with the inhaler, labeling, and packaging, including reading the IFU if they chose to do so. The goal of Task 1 was to assess intuitiveness and first use of the inhaler. For Tasks 2 and 3, the subject repeated Task 1 twice more with the same inhaler, with the goal of Task 2 being to show any improvement in technique, and of Task 3, to demonstrate learned “at-home use.” If the subject used the Inhub inhaler in the horizontal position during any of the three tasks, he or she was asked to repeat the inhalation in the horizontal position with an instrumented Inhub inhaler to record his or her inhalation profile. If the inhalation profile indicated that the peak inhalation flow rate (PIFR) was ≥30 L/min and the total inhaled volume (TIV) was ≥1 L, it was concluded that, based on an understanding of patient inhalation profiles for this indication and the pharmaceutical performance of the inhaler, in real use, the subject would have received a therapeutic dose. If not, an “orientation-related use error” was recorded and the root cause was identified.

If the subject was a current Advair Diskus user, he or she was asked to use the empty Diskus inhaler three times (Tasks 4–6); Task 4 was meant to demonstrate any prelearned misuse behaviors with the Advair Diskus, and Tasks 5 and 6 were meant to confirm the misuse behaviors. Based on the four key operating steps of the Inhub IFU (open, push down, inhale, and close), performance-based data were collected against predefined usability criteria. The outcomes against the usability criteria were recorded as success, issues but recovered, or fail; postsession interviews were conducted to determine the root cause of any action that was not recorded as success. Table 1 details each task, along with the success criteria per task for both the Inhub and Diskus inhalers.

**Table 1.** Success Criteria for Inhub and Diskus Inhaler Tasks for Study 1

Task	Inhaler	Script printed on task card	Purpose of task	Criteria assessed^a^
1	Inhub	Current Advair Diskus subjects only: “You have been using Advair for a number of years. However, during your last visit to the pharmacy, you were given this inhaler as a generic alternative. The medication and dose frequency are exactly the same as they were with Advair; 1 dose twice a day, morning and evening. The only difference is the inhaler. This is now your first day of using the inhaler. Prepare to take the medication and take a dose.” Asthma and/or COPD subjects naïve to all dry powder inhalers “You have been using a metered dose inhaler for a number of years. However, the doctor has just prescribed a dry powder inhaler which is designed to control your asthma. You will take the medication twice a day, once in the morning and once in the evening. You took the prescription to the pharmacy and were given this inhaler. This is your first day of using the inhaler. Prepare to take the medication and take a dose.”	Assess the intuitiveness and first use of the inhaler	1–2, 3i, and 4–6
2	Inhub	“You are now due to take your next dose using the same inhaler. Prepare to take the medication and take your next dose.”	Show any improvement in technique	1–2, 3i, and 4–6
3	Inhub	“You are now due to take your next dose using the same inhaler. Prepare to take the medication and take your next dose.”	Demonstrate “at-home use”	1–2, 3i, and 4–6
Any subject who holds the Inhub inhaler horizontally for the inhalation step in any of the first 3 tasks will now be asked to demonstrate his or her inhalation horizontally through an instrumented Inhub inhaler, and his or her inhalation profile will be recorded.				3ii
4	Diskus	Current Advair Diskus subjects only: “Please take a dose (using this inhaler) as you would do normally with your Advair Diskus.”	Demonstrate any pre-learned misuse behaviors with the Advair Diskus	1–6
5	Diskus	Current Advair Diskus subjects only: “Please take another dose (using this inhaler) as you would do normally with your Advair Diskus.”	Confirm any pre-learned misuse behaviors from their use of Advair Diskus	1–6
6	Diskus	Current Advair Diskus subjects only: “Please take another dose (using this inhaler) as you would do normally with your Advair Diskus.”	Confirm any pre-learned misuse behaviors from their use of Advair Diskus	1–6

^a^ Criteria: (1) Subject can open the mouthpiece cover; (2) Subject pushes the lever as far as it will go; (3i) Subject holds the inhaler in the vertical orientation – OR – (3ii) If the subject holds in the horizontal orientation, inhaled flow rates of ≥30 L/min and inhaled volumes of ≥1 L are achieved; (4) Subject leaves the lever fully pushed during inhalation; (5) Subject fully closes the mouthpiece cover for storage; and (6) Subject is not injured during use of the inhaler.

### Study 2: assessment of robustness of Inhub inhaler

#### Study design and conduct

An open-label study (EudraCT: 2015-000463-13) was performed to assess the robustness of the Inhub inhaler. The robustness assessment was made by evaluating the *in vitro* pharmaceutical performance of FP and salmeterol remaining in the Inhub inhaler after BID oral inhalation of Wixela Inhub (250 μg FP/50 μg salmeterol xinafoate) by subjects with asthma or COPD for 21.5 (±3) days. The study protocol and other relevant study documentation were reviewed and approved by the applicable regulatory authority and an independent ethics committee. Study 2 was conducted at two investigator sites in accordance with the requirements of the Declaration of Helsinki and applicable local regulatory requirements, and all subjects (and their legal guardian, as appropriate, if the subject was aged 12–15 years) provided written informed consent.

#### Study subjects

Study 2 enrolled subjects diagnosed with asthma or COPD. Subjects with asthma were required to have a screening prebronchodilator forced expiratory volume in 1 second (FEV_1_) of ≥50% of predicted value and be ≥12 years of age. Exclusion criteria for subjects with asthma included evidence of active, severe, progressive, and/or uncontrolled clinical disease other than asthma; significant disease instability/uncontrolled asthma or history of life-threatening asthma; or use of any medication contraindicated in the Advair Diskus label. Subjects with COPD were required to have a screening postbronchodilator FEV_1_ ≥40% of predicted value, have an FEV_1_/forced vital capacity (FVC) ratio of <0.7, and be ≥40 years of age. Exclusion criteria for subjects with COPD included alpha-1 antitrypsin deficiency, other chronic or active respiratory disorder, symptoms of or treatment for acute exacerbations of COPD that required antibiotics and/or oral/systemic corticosteroids, inpatient hospitalization during the 28 days preceding Visit 1 (screening) or during the period before Visit 2 (day 1), or use of any medication contraindicated in the Advair Diskus label.

#### Study assessments

Study 2 involved three visits to the clinic: Visit 1 (screening) for consent/screening procedures, Visit 2 (Day 1) for start of dosing, and Visit 3 (day 22 [ ± 3]) for end of dosing, return of the Inhub inhaler, and follow-up activities. Visit 1 was conducted a maximum of 28 days before the first dose of study medication (i.e., Visit 2), although Visits 1 and 2 could be combined into a single visit if practical (e.g., if any restricted medications had been withheld for the appropriate period of time). At Visit 2, the investigator provided inhaler training and observed dosing. The investigator recorded any significant deviation in the dosing procedure, and additional training/corrective action was given. For Visit 3, study medication was dosed and observed in the clinic, with any significant deviation in the dosing procedure recorded by the investigator. Subjects also underwent safety discharge procedures (physical examination, vital signs [blood pressure and pulse rate], electrocardiogram, and spirometry), with any clinically significant changes being recorded as adverse events (AEs). The subjects reported to the investigator any AEs occurring up to 30 days after the last dose of medication from Wixela Inhub. The study (from screening to discharge) was 3–8 weeks in duration for each subject.

Subjects continued to receive their previous asthma or COPD medication (including maintenance and rescue medications) throughout the study, although subjects previously taking ICSs, LABAs, or ICSs/LABA fixed-dose combinations ceased taking that particular drug on the day before their first dose of study medication.

Subjects were required to take a dose of study medication from Wixela Inhub in the morning and evening (∼12 hours apart) as outpatients. Subjects recorded the date and time of study medication dosing in a diary between study visits; subjects also recorded any issues related to their use of the inhaler. The subjects were contacted by the investigator by telephone for an interim check-in (on day 8 [ ± 3]) to collect any AEs and remind subjects of compliance requirements.

The investigator evaluated safety using physical examination, 12-lead electrocardiogram, vital signs (blood pressure and pulse rate), spirometry (FEV_1_ and FVC), with any clinically significant changes recorded as AEs. Subjects were routinely queried for AEs using open-ended questions. Spontaneously reported AEs were also recorded. Subjects were monitored for any AEs from the signing of the informed consent forms to 30 days after the last dose of study medication.

#### *In vitro* testing

At Visit 3, inhalers were returned to the investigator and then sent to the testing laboratory within 7 days of the subject's last dose. The number of returned inhalers suitable for testing was recorded along with a summary of all inhalers deemed not suitable (including the reason why). Suitable inhalers were utilized for *in vitro* pharmaceutical performance testing if subjects had ≥16 days of BID dosing confirmed through dose counters (i.e., the dose counter read ≤28 from a starting dose counter number of 60). This equated to ≥75% compliance with per-protocol dosing. Upon receipt at the testing laboratory, the external surface of all inhalers was wiped using an alcohol swab to remove saliva and contamination. An external visual inspection was conducted to record any observations of damage or defect and determine the suitability of the inhaler for testing. Before testing, samples were stored under monitored/controlled laboratory conditions. All inhalers were tested within 60 days of initial removal from the pouch in which they were supplied.

Robustness of the Inhub inhaler was assessed by performing *in vitro* tests for drug content (assay), degradation products, microbiology, water content, delivered dose uniformity and aerodynamic particle size distribution (APSD), assay (drug content), and degradation product (organic impurities) tests on the drug remaining in unused pockets in the inhaler. The number of inhalers assessed by each test is given in Table 2.

**Table 2.** Sample Size and Requirements for *In Vitro* Testing for Study 2

Test	No. of inhalers required	No. of pockets tested per inhaler	
Microbiology	40	Sufficient inhalers/pockets opened to deliver 5 g of powder	Conducted and reported in line with the harmonized pharmacopeia-defined method for inhaled products (USP). Disks were removed from the used inhalers and externally wiped with an alcohol swab before removal of powder for microbiological assessment
Delivered dose uniformity/APSD	30	1	One pocket from each of 30 inhalers was tested for emitted dose and APSD (at 60 L/min)
Assay/degradation products	10	≤7	One pocket from each of 10 inhalers
Water content	4	≤7	Water content: samples taken from at least four inhalers
Analytical retest samples	15	Test dependent	Inhalers retained for laboratory investigations of aberrant results. Sample size based on retest requirement of any individual test from assay/degradation products/water content

The same inhaler could be used for more than one test type.

APSD, aerodynamic particle size distribution; USP, United States Pharmacopeia.

The test for APSD was described as *Test 2* in the United States Pharmacopeia (USP) product monograph.^(15)^ APSD was determined at 60 L/min using USP <601> Apparatus 5 (Next Generation Impactor™ [NGI]; Copley, Nottingham, United Kingdom). A vacuum pump and a critical flow controller (Copley TPK) were used. The flow rate was measured as described in USP <601>, using a flowmeter calibrated for the volumetric flow leaving the meter (Copley DFM3 or equivalent). Flow duration was set to give a volume of 4 L. The FP and salmeterol were recovered from the NGI stages and accessories (mouthpiece adapter/induction port and preseparator) with a methanol buffer diluent and were analyzed by liquid chromatography.

The delivered dose uniformity test was carried out at 60 L/min using USP <601>Apparatus B (Dosage Unit Sampling Apparatus; Copley, Nottingham, United Kingdom). A vacuum pump and a critical flow controller (Copley TPK) were used. The flow rate was measured as described in USP <601>, using a flowmeter calibrated for the volumetric flow leaving the meter (Copley DFM or equivalent). Flow duration was set to give a volume of 2 L.

The tests for delivered dose uniformity and drug content used the same diluent and chromatographic conditions as the USP monograph APSD *Test 2* and have been validated for specificity, linearity, accuracy, precision, and chromatographic robustness. In addition, the drug content assay was shown to give equivalent results (within ±1%) of those obtained from the corresponding USP monograph test (which uses the same chromatographic conditions as the USP monograph APSD *Test 1*).

The test for degradation products used liquid chromatography with UV detection and was validated for specificity, linearity, accuracy, precision, and chromatographic robustness. Owing to differences in assignment and quantification of impurities, this method does not generate results that are comparable with those in the corresponding USP monograph test for organic impurities.

Tests for total aerobic microbial and yeast content were carried out according to USP <61>. The absence of specified microorganisms was tested according to USP <62>. *Pseudomonas aeruginosa*, *Staphylococcus aureus*, and bile-tolerant gram-negative organisms were tested, as per USP <1111>: *Microbiological Examination of Nonsterile Products: Acceptance Criteria for Pharmaceutical Preparations, and Substances for Pharmaceutical Use*.

Water content testing was carried out by Karl–Fischer titration.

### Statistical analyses

Neither study generated data that underwent formal statistical analysis.

#### Study 1

The minimum sample size was set at 15 subjects per user group (six groups total: three age groups [≥12 to <18, ≥18 to <65, and ≥65 years] each for DPI-naive and Advair Diskus users). This sample size was based on the research completed by Faulkner et al.^(16,17)^ that suggested a sample of 15 subjects was sufficient to find a minimum of 90% and an average of 97% of all known problems in user testing studies. The adult group consisted of 25 subjects, whereas the elderly and adolescent groups consisted of 15 subjects each. Based on IMS Health (now part of IQVIA) data^(18)^ from January 2013 to December 2013, only 4% of all U.S. subjects using Advair Diskus were adolescents (aged 12–17 years); the ratio of elderly to adult Advair Diskus users was 38:62. Therefore, using those criteria, the adolescent and elderly groups consisted of the minimum sample size.

#### Study 2

Approximately110 subjects with asthma/COPD were planned to be recruited and receive Wixela Inhub 250/50. The sample size of 110 subjects was selected on the basis of the number of inhalers required for each *in vitro* test of pharmaceutical performance (the sample size required for testing was 95), and the assumption that some subjects would drop out of the study and that not all inhalers would be returned or be suitable for testing. To ensure that a broad population of subjects provided used inhalers, asthma subjects were aged ≥12 years, and ∼10–20 subjects were to be recruited from the 12- to 17-year age group. To adequately represent elderly subjects, ∼10–30 subjects were to be recruited from the ≥65-year age group. Two population sets were described in Study 2: the enrolled set, consisting of all subjects who signed an informed consent form, and the safety analysis set, consisting of all subjects who received ≥1 dose of study drug.

## Results

### Study 1: usability validation study of Inhub inhaler

#### Subjects

**FIG. 2.**
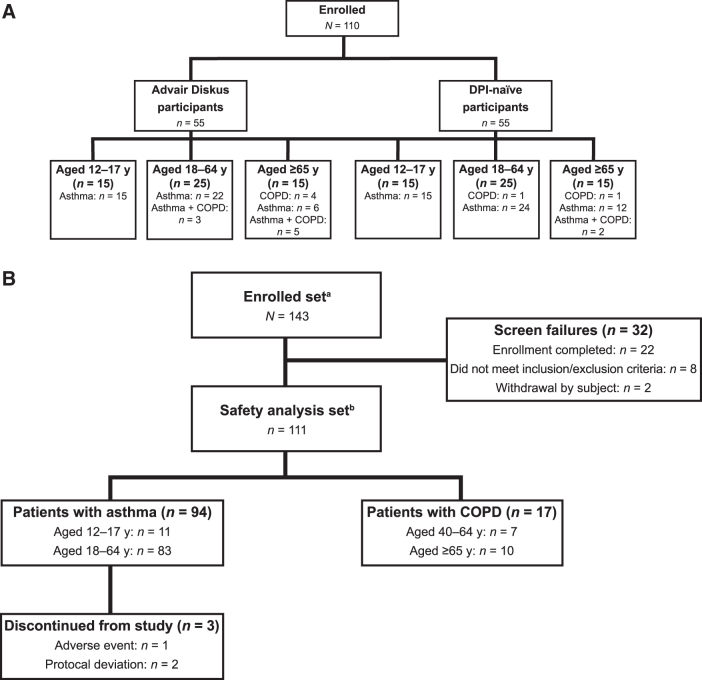
Patient disposition for **(A)** Study 1 and **(B)** Study 2. ^a^Enrolled set defined as all subjects who signed an informed consent form. ^b^Safety analysis set defined as all subjects who received ≥1 dose of study drug. COPD, chronic obstructive pulmonary disease; DPI, dry powder inhaler; y, years.

Study 1 enrolled 110 subjects, and all completed the study (Fig. 2A). The population was evenly divided between subjects who were either Advair Diskus users (*n* = 55) or DPI naive (*n* = 55) (Table 3).

**Table 3.** Baseline Demographics—Study 1

Condition	Category	Advair Diskus users,* n* = 55(%)	DPI naive,* n* = 55(%)
Age (years)	≥12 to <18 years	15 (27.3)	15 (27.3)
	≥18 to <65 years	25 (45.5)	25 (45.5)
	≥65 years	15 (27.3)	15 (27.3)
Gender	Male	29 (52.7)	28 (50.9)
	Female	26 (47.3)	27 (49.1)
Respiratory condition	Asthma	43 (78.2)	51 (92.7)
	COPD	4 (7.3)	2 (3.6)
	Asthma/COPD	8 (14.5)	2 (3.6)
Literacy^a^	Grades 2–5	3 (5.5)	3 (5.5)
	Grades 6–8	14 (25.5)	11 (20.0)
	Grades 9–12	38 (69.1)	41 (74.5)
Manual dexterity issues	No	45 (81.8)	49 (89.1)
	Yes	10 (18.2)	6 (10.9)
Dominant hand	Right	53 (96.4)	46 (83.6)
	Left	2 (3.6)	7 (12.7)
	Ambidextrous	0	2 (3.6)

^a^ Grade level equivalent literacy score.

DPI, dry powder inhaler.

#### Evaluation of Inhub device usability

A summary of Tasks 1, 2, and 3 completion rates (all subjects using Inhub inhaler; third inhalation simulating “at-home use”), including success rates and any failures, is given in Table 4. With regard to learned “at-home” use, all subjects were able to complete Task 3A (opening mouthpiece cover) successfully. For Task 3B (pushing lever as far as it goes), the success rate was 108/110 (98.2%). One subject (aged ≥65 years; DPI naive) committed an error (did not push the lever all the way down). This subject attempted to use the mouthpiece cover as a handle to secure the device in her hand while simultaneously pushing down the yellow lever. The opposing force caused the cover to partially close while she was moving the lever, resulting in the lever not being pushed all the way down to the purple arrows. Because the mouthpiece cover is not designed to be used as a handle, this is a misuse scenario. Another subject (aged ≥65 years; Advair Diskus user) initially did not push the lever down, but noticed the error on his or her own and corrected this error. This subject indicated that if the device had contained actual medication, he or she may have known earlier that the lever was not pushed down fully and, therefore, that the medication was not available during the initial inhalation.

**Table 4.** Tasks 1–3 (Inhub Inhaler) and Tasks 4–6 (Diskus Inhaler) Success Rates for Study 1

Task and usability criteria	Success (no review required) (%)	Failure (review required)	
		Issues but recovered (%)	Error (%)
Task 1—First Inhub dose			
1A: User can open the mouthpiece cover	108/110 (98)	2/110 (2)	0/110 (0)
1B: User pushes lever as far as it will go	107/110 (97)	2/110 (2)	1/110 (1)
1C: User leaves the lever fully pushed during inhalation	105/110 (95)	1/110 (1)	4/110 (4)
1D: User holds the inhaler in the vertical orientation	99/110 (90)	0/110 (0)	11/110 (10)
1E: User fully closes the mouthpiece cover for storage	109/110 (99)	0/110 (0)	1/110 (1)
Task 2—Second Inhub dose			
2A: User can open the mouthpiece cover	110/110 (100)	0/110 (0)	0/110 (0)
2B: User pushes lever as far as it will go	105/110 (95)	2/110 (2)	3/110 (3)
2C: User leaves the lever fully pushed during inhalation	107/110 (97)	0/110 (0)	3/110 (3)
2D: User holds the inhaler in the vertical orientation	100/110 (91)	0/110 (0)	10/110 (9)
2E: User fully closes the mouthpiece cover for storage	110/110 (100)	0/110 (0)	0/110 (0)
Task 3—Third Inhub dose			
3A: User can open the mouthpiece cover	110/110 (100)	0/110 (0)	0/110 (0)
3B: User pushes lever as far as it will go	108/110 (98)	1/110 (1)	1/110 (1)
3C: User leaves the lever fully pushed during inhalation	108/110 (98)	0/110 (0)	2/110 (2)
3D: User holds the inhaler in the vertical orientation	100/110 (91)	0/110 (0)	10/110 (9)
3ii: User achieves an inhaled flow rate of ≥30 L/min and total inhaled volume of ≥1 L/min^a^	9/10 (90)	0/10 (0)	1/10 (10)
3E: User fully closes the mouthpiece cover for storage	110/110 (100)	0/110 (0)	0/110 (0)
Task 4—First Diskus dose			
4A: User can open the mouthpiece cover	55/55 (100)	0/55 (0)	0/55 (0)
4B: User pushes lever as far as it will go	55/55 (100)	0/55 (0)	0/55 (0)
4C: User leaves the lever fully pushed during inhalation	46/55 (84)	0/55 (0)	9/55 (16)
4D: User holds the inhaler in the horizontal orientation	53/55 (96)	0/55 (0)	2/55 (4)
4E: User fully closes the mouthpiece cover for storage	50/55 (91)	0/55 (0)	5/55 (9)
Task 5—Second Diskus dose			
5A: User can open the mouthpiece cover	48/48 (100)	0/48 (0)	0/48 (0)
5B: User pushes lever as far as it will go	52/52 (100)	0/52 (0)	0/52 (0)
5C: User leaves the lever fully pushed during inhalation	42/52 (81)	0/52 (0)	10/52 (19)
5D: User holds the inhaler in the horizontal orientation	50/52 (96)	0/52 (0)	2/52 (4)
5E: User fully closes the mouthpiece cover for storage	47/52 (90)	0/52 (0)	5/52 (10)
Task 6—Third Diskus dose			
6A: User can open the mouthpiece cover	46/46 (100)	0/46 (0)	0/46 (0)
6B: User pushes lever as far as it will go	51/51 (100)	0/51 (0)	0/51 (0)
6C: User leaves the lever fully pushed during inhalation	42/51 (82)	0/51 (0)	9/51 (18)
6D: User holds the inhaler in the horizontal orientation	49/51 (96)	0/51 (0)	2/51 (4)
6E: User fully closes the mouthpiece cover for storage	46/51 (90)	0/51 (0)	5/51 (10)

^a^ For the 10 subjects who inhaled with the Inhub in the horizontal orientation.

For Task 3C (leaves lever fully pushed during inhalation), two subjects (both pediatric: one DPI naive and one Advair Diskus user) committed errors. The DPI-naive subject's error was seemingly related to confusion on the part of the parent who read the IFU and miscommunicated the instructions to the subject. The IFU's instructions regarding device operation were initially unclear to the subject's mother, who explained to the subject how to use the device. She was relying on her interpretation of the IFU steps, which was incorrect. The subject's mother indicated that she was thinking about how the subject's other inhalers (all pMDIs) worked while she was reading the IFU. The Advair Diskus user's error was related to her behavior that indicated she preferred to do it “my way,” regardless of correctness. The subject knew the correct inhalation technique but indicated that she consciously chose to “do it my way” because it was “easier” for her. The subject reported that she used her Advair Diskus inhaler in a way other than described in the IFU as well, despite understanding what was required. It was noted throughout the session that this subject demonstrated behaviors indicative of a potential social and/or psychological disorder. This was not an exclusion for recruitment, however, and the subject was included in the study.

Task 3D, related to holding the Inhub inhaler device in the vertical orientation, was successfully completed by 100 of the 110 subjects (90.9%), with 10 subjects inhaling with the Inhub inhaler in the horizontal orientation. These 10 subjects comprised one DPI-naive subject (aged ≥18 to <65 years) and nine Advair Diskus users (two aged ≥12 to <18 years, four aged ≥18 to <65 years, and three aged ≥65 years). Of these, nine subjects (90.0%) still achieved a successful inhalation based on the indicated criteria, demonstrating that they would have received an efficacious dose despite horizontal inhaler orientation. The only subject who did not achieve a successful inhalation (aged ≥65 years; Advair Diskus user) had been initially classified as a “device error”; however, it was demonstrated that poor inhaled volumes occurred whether the subject held the device in the horizontal (PIFR, 14.4 L/min; TIV, 0.48 L) or vertical (PIFR, 19.1 L/min; TIV 0.69 L) orientation, indicating that orientation alone was not the cause of her poor inhaled volumes. The cause of the error was thus attributed to poor lung function. All subjects successfully completed the final task (Task 3E, fully closing the mouthpiece cover for storage).

All Advair Diskus users were then asked to perform three inhalations with an empty Advair Diskus. It should be noted that the following percentages reported are not all out of 55. Users were omitted if they chose not to complete the task or if they could not complete the task because of a previous failure (e.g., user left the mouthpiece cover open on the previous task and, therefore, could not open it on the task at hand). Upon the third inhalation with the Diskus (Task 6), subjects demonstrated 100% success with Tasks 6A (opening the mouthpiece cover) and 6B (pushing lever as far as it will go). Only 42 of 51 Advair Diskus users (82.4%) were successful at Task 6C (leaving lever fully pushed during inhalation) and 46 of 51 (90.2%) at Task 6E (fully closing mouthpiece cover for storage). For Task 6C, subjects most frequently indicated that they had “always done it that way,” and for Task 6E, subjects indicated that they did not close the mouthpiece cover because they were not aware that it had a cover, because they did not normally cover it, or because they believed that they would be asked to use it again.

For Task 6D, related to the use of the Advair Diskus in the horizontal orientation, 49 of the 51 subjects (96.1%) who performed the third Diskus inhalation successfully used the device in the horizontal orientation. Two subjects (both pediatric) held the Advair Diskus in the vertical position. In both cases, the subjects consciously chose to use a different technique (i.e., the subjects were aware they were using it incorrectly but nevertheless persisted in that behavior).

Negative transfer of behaviors from routine use of Advair Diskus was seen in the interaction with the Inhub inhaler in a number of subjects with one subject inhaling at the same time as pushing the lever as they did with their current Diskus inhaler and nine subjects relying on familiarity with the orientation of the use of the Diskus inhaler to direct them to hold Inhub in the horizontal orientation. Robust design verification and risk management through the development of the Inhub inhaler confirm that such deviations from intended use will not lead to unacceptable harm to the user. Not all misuse was transferred to the use of Inhub, as can be seen by the 100% success in users closing the Inhub mouthpiece cover for storage, which can be attributed to the device design.

### Study 2: assessment of robustness of Inhub inhaler

#### Subjects

In Study 2, 111 of 143 enrolled subjects received ≥1 dose of study drug and were included in the safety analysis set (Fig. 2B). The baseline demographics are given in Table 5. Of these 111 subjects (asthma, *n* = 94; COPD, *n* = 17), 108 (97.3%) completed the study. Three subjects discontinued the study: two because of a protocol deviation (both left the country during the study and could not attend study visits) and one because of an AE (atrial fibrillation, mild in intensity) considered by the investigator to be unlikely related to study drug. Wixela was administered for 21.5 (±3) days, with the last dose in the clinic at Visit 3. The mean (standard deviation) and median (range) dose counter number after dosing at Visit 3 for all subjects were 18.3 (2.93) (Table 6) and 18.0 (11–40), respectively. All subjects who completed the study had ≥75% compliance with per-protocol dosing.

**Table 5.** Baseline Demographics for Study 2

	No. of subjects (*N = *111)^a^
Disorder, *n* (%)	
Asthma	94 (84.7)
COPD	17 (15.3)
Age (years)	
Mean	41.4
SD	16.3
Min	12
Median	40.0
Max	76
Age category (years), *n* (%)	
Asthma 12–17	11 (9.9)^b^
Asthma ≥18 to <65	83 (74.8)^b^
Asthma ≥65	0^b^
COPD ≥40 to <65	7 (6.3)^c^
COPD ≥65	10 (9.0)^c^
Gender, *n* (%)	
Male	63 (56.8)
Female	48 (43.2)
Race, *n* (%)	
Asian	3 (2.7)
Black or African American	4 (3.6)
White	103 (92.8)
Other/mixed	1 (0.9)

^a^ Safety analysis set.

^b^ Subjects diagnosed with asthma.

^c^ Subjects diagnosed with COPD.

min, minimum; max, maximum; SD, standard deviation.

**Table 6.** Returned Inhalers, Dosing Compliance, and Suitability for *In Vitro* Testing for Study 2

	Wixela Inhub 250/50 BID (*n = *111)
No. of inhalers dispensed, *n* (%)	111 (100)
No. of inhalers returned, *n* (%)	111 (100)
No. of dose counters after dosing at Visit 3, mean (SD)	18.3 (2.93)
Compliance percentage, mean (SD)	99.8 (1.66)
Compliance category, *n* (%)	
<75% of per-protocol dosing	0
≥75% of per-protocol dosing	111 (100)
Inhalers unsuitable/unavailable for testing, *n*^a^	4
Inhalers available for testing, *n*^b^	107
Inhalers utilized for testing, *n*^c^	84

^a^ Includes two inhalers for subjects who discontinued before Visit 3 and two inhalers with unsubstantiated device complaint investigations.

^b^ Includes 13 inhalers only suitable for water content assessment.

^c^ The total number of inhalers required for testing was 84; the additional 23 inhalers were not used for *in vitro* testing.

BID, twice daily.

#### *In vitro* testing of pharmaceutical performance

A total of 107 inhalers were suitable and available for *in vitro* testing of pharmaceutical performance (Table 6). Of these 107 inhalers, 13 underwent a temperature excursion during transport and were, therefore, deemed only suitable for water content assessment (2 of these inhalers were included in the water content assessment, the other 11 inhalers were not used for any testing as there was a sufficient number of inhalers for all the tests). The total number of inhalers required for testing was 84; hence, this number of inhalers was assigned for *in vitro* testing of pharmaceutical performance testing, and the additional 23 inhalers were not used for any testing.

Results of *in vitro* testing for pharmaceutical performance are given in Table 7. Key tests indicative of the physicochemical and microbiological integrity of the inhalers were carried out. In Table 7, the results are compared (where possible) with the USP monograph for FP and salmeterol xinafoate inhalation powder, which is the applicable public standard for the approved product.^(15)^

**Table 7.** Results of *In Vitro* Testing for Pharmaceutical Performance for Study 2 Compared with United States Pharmacopeia Monograph Acceptance Criteria

Test	Result	USP monograph acceptance criteria	
Assay (mean drug content, percentage of label claim)			
Fluticasone propionate	97.6	90.0–110.0	
Salmeterol	97.8	90.0–110.0	
Aerodynamic particle size distribution (USP monograph Test 2)^a^		USP monograph Table 3	USP monograph Table 4
Fluticasone propionate (range, μg/actuation)			
Sum of mouthpiece adapter, induction port, preseparator, Stage 1, and Stage 2 (Group 1)	164–198	158–232	142–255
Sum of Stages 3–7 and MOC (Group 2)	41–58	27–64	24–70
Sum of Stages 4 and 5 (Group 3)	23–35	13–38	12–42
Sum of Stage 6, Stage 7, and MOC (Group 4)	1–3	NMT 3	NMT 3
Salmeterol (range, μg/actuation)			
Sum of mouthpiece adapter, induction port, preseparator, Stage 1, and Stage 2 (Group 1)	37–41	36–48	32–53
Sum of Stages 3–7 and MOC (Group 2)	6–9	4–10	4–11
Sum of Stages 4 and 5 (Group 3)	3–5	2–6	2–7
Sum of Stage 6, Stage 7, and MOC (Group 4)	0.1–0.3	NMT 0.5	NMT 0.6
Delivered dose uniformity (mean [range] as percentage of target emitted dose)			
Fluticasone propionate	97 (92–104)	Mean is between 85% and 115% of target emitted dose • NMT 3 of 30 doses are outside 80%–120% of target emitted dose • No dose is outside 75%–125% of target emitted dose	
Salmeterol	103 (98–109)		
Degradation products	NMT 0.10%	Not applicable	
Microbial enumeration tests (USP <61>)			
Total aerobic microbial count	<10 cfu/g	NMT 10^2^ cfu/g (200 cfu/g)	
Total combined yeasts/molds count	<10 cfu/g	NMT 10 cfu/g (20 cfu/g)	
Specified microorganisms (USP <62>)			
*Pseudomonas aeruginosa*	Absent	Absent	
*Staphylococcus aureus*			
Bile-tolerant gram-negative organisms			
		
		
		
Water content, %	5	Not applicable	
		

^a^ The requirements for the masses of fluticasone propionate and salmeterol deposited in each grouping of the sampling apparatus for each inhaler are given in USP monograph Table 3. The article meets the requirements if NMT one of six inhalers fails to meet the requirements in USP monograph Table 3 but meets the requirements in USP monograph Table 4.

MOC, Micro-orifice collector; NMT, not more than.

The USP monograph for FP and salmeterol xinafoate inhalation powder first became official after the robustness study was completed. The USP monograph was subsequently revised to include the test for APSD used in the robustness study. The other tests used in the study were in-house tests, of which those for drug content and delivered dose uniformity generate results equivalent to the corresponding tests in the USP product monograph.

All inhalers tested demonstrated conformity with the USP monograph specification in respect of assay, delivered dose uniformity, and APSD. One inhaler gave an aberrant APSD result below the range given in Table 7 for the stage groupings indicated, and outside the USP monograph limits for the sum of mouthpiece adapter, induction port, preseparator, Stage 1, and Stage 2 for both FP and salmeterol. The mass balance associated with this determination fell outside the range specified in the USP general chapter <601>. On investigation, no laboratory error was found. A further six doses from the same inhaler were tested, all of which gave results within the stated range and within the USP monograph limits. Although the original result was not invalidated, it was concluded that the original result was not reflective of the device or the batch.

Microbiological testing was carried out for the total aerobic microbial count, total combined yeasts/molds count, and the absence of specified organisms *P. aeruginosa*, *S. aureus*, and bile-tolerant gram-negative organisms, demonstrating conformance with the USP general chapter <1111>.

No chemical degradation of the active ingredients was observed in the returned inhalers. The moisture content was 5%, as expected considering the water content of lactose monohydrate^(19)^ (which constitutes >97% of the powder formulation), demonstrating that the device was robust to water ingress during use.

Two inhaler complaints were made during the study, but after further investigation, both were deemed unsubstantiated as the inhalers showed no defects and had performed as expected upon investigation. No inhaler failures were confirmed during the study.

#### Safety assessment

Table 8 presents an overall summary of subjects who experienced ≥1 AEs. Among the 111 subjects in the safety set, 34 subjects (30.6%) had a total of 55 treatment-emergent AEs (TEAEs). Only one subject had a TEAE that led to study discontinuation (mild atrial fibrillation that the investigator considered unlikely to be related to study drug). Thirty-four subjects (30.6%) had mild AEs, one subject (0.9%) had a moderate AE (oral candidiasis), and none had severe AEs. The most common TEAE was headache, which was reported in 11 subjects (9.9%). No deaths, pregnancies, or serious TEAEs occurred during the study.

**Table 8.** Treatment-Emergent Adverse Events Occurring in ≥2 Subjects in Study 2

Preferred term,* n *(%)	Wixela Inhub 250/50 BID (*n = 111*)
No. of subjects with ≥1 TEAEs	34 (30.6)
Headache	11 (9.9)
Dysphonia	4 (3.6)
Oropharyngeal pain	4 (3.6)
Cough	2 (1.8)
Dyspepsia	2 (1.8)
Pharyngitis	2 (1.8)
Rhinitis	2 (1.8)
Upper respiratory tract infection	2 (1.8)
Wheezing	2 (1.8)

BID, twice daily; TEAE, treatment-emergent adverse event.

## Discussion

A large body of data exists to support the importance of correct inhaler device usage in the efficacy of inhaled medications for subjects with asthma and COPD. Correct use of an inhaler device is highly correlated with better disease control and, conversely, improper device usage is more common in those with poorly controlled disease.^(20–22)^ Therefore, concerns that subjects who are accustomed to using one commercial device should be able to use an equivalent generic device safely and effectively are not trivial given the potential for lack of training and substitutability at the pharmacy counter. The Wixela Inhub has been designed using a wealth of expertise in ergonomics and industrial design and thoroughly tested to ensure that subjects will be able to use it by following the provided IFU, even if they are not instructed by the prescriber or another health care provider.

The focus on human factors and orientation of use in Study 1 confirm that the key operational steps of the Inhub inhaler are similar to that of the Diskus inhaler such that current Diskus users could demonstrate safe and effective use of the Inhub inhaler and easily switch between devices and that the Inhub inhaler is intuitive to use such that users across the demographics of the intended user population could demonstrate safe and effective use.^(13)^ Regardless of the difference in intended orientation of the inhalers, most subjects (100/110) held the Inhub inhaler in the correct orientation; of those who did not hold the Inhub inhaler in the correct orientation (orientation-related use error), all except one (subject's error was attributed to poor lung function and thus likely would not have received the appropriate dose with Diskus inhaler either) still achieved a PIFR of ≥30 L/min and a TIV of ≥1 L. Study 1 thus demonstrated that subjects could use the inhaler as intended and, in particular, that there was a low likelihood of a device use error that could potentially lead to a patient receiving a subtherapeutic dose.

The results of Study 2 confirm the robustness of the Inhub inhaler. After ∼3 weeks (21 ± 3 days of dosing) of regular use of the Inhub inhaler by pediatric, adult, and elderly subjects with asthma or COPD in an outpatient setting, comprehensive *in vitro* testing demonstrated that the FP and salmeterol pharmaceutical performance were preserved.

The combined outcome of both studies demonstrate that the Inhub device can be successfully used by the intended user population to deliver a therapeutic dose safely and effectively with no impact of user handling on functional and pharmaceutical performance after >70% use of the device. This outcome satisfies design validation of both the inhaler and associated IFU and meets FDA requirements for a generic FP/salmeterol xinafoate inhalation device.

## Notes

Reviewed by:

Guenther Hochhaus
